# Brief History of Opioids in Perioperative and Periprocedural Medicine to Inform the Future

**DOI:** 10.31486/toj.22.0065

**Published:** 2023

**Authors:** Philip G. Boysen, Jenilkumar H. Patel, Angelle N. King

**Affiliations:** ^1^ Emeritus Professor and Chair, The University of North Carolina at Chapel Hill, Chapel Hill, NC; ^2^ Tulane University School of Medicine, New Orleans, LA

**Keywords:** *Analgesics–non-narcotic*, *analgesics–opioid*, *opioid-related disorders*, *receptors–opioid*

## Abstract

**Background:** Opioids and derivatives of opium had been used as analgesics for thousands of years before the introduction of inhalational anesthetic agents. Once these early volatile agents were in widespread use, opioids were used as part of anesthetic care for premedication, as intraoperative adjuncts to general anesthesia, and for the management of postoperative pain. Evidence of growing dependence on opioids in the perioperative and periprocedural patient is supported by the ongoing research to develop synthetic opioids and to customize the pharmacokinetics and pharmacodynamics to achieve specific therapeutic goals.

**Methods:** We explore the history of opioid use in perioperative care as a means of future management in light of new persistent opioid abuse.

**Results:** As the opium chemical structure has been modified, newer nonopioid analgesics have been approved and brought into clinical practice. Opioid-sparing and opioid-free anesthetic techniques are not only a possibility, but a reality.

**Conclusion:** Continuing research in neurobiology and addiction genetics will ultimately lead to a pharmacogenetic approach to patients at risk for new persistent opioid abuse.

## INTRODUCTION

Opioids had been used for pain relief for thousands of years before William Morton demonstrated the effects of inhalational anesthesia in 1846.^1-3^ The leap from analgesia to anesthesia temporarily reduced the clinical use of opioids as analgesics in the United Kingdom and the United States. However, opioids were soon once again included in the practice of anesthesiology but as an adjunct to inhalational agents.^4^

Given the current environment in North America where opioid abuse has been recognized as an epidemic, reviewing the history of opioid use in clinical medicine and anesthesiology is useful for informing the future of clinical practice. In a large multidisciplinary study published in 2017, the incidence of new persistent opioid abuse following surgery and other procedures was reported to be 6%.^5^ Some individuals struggle with opioid abuse following seemingly minor procedures and only a single exposure to the drug.^5^

## OPIOIDS IN THE 19TH CENTURY

In addition to Morton's ether demonstration in 1846, other occurrences in the 19th century are of historic importance. Friedrich Sertürner isolated morphine from opium in 1803.^6^ Codeine (methylmorphine), molecular formula C18H21NO3, was isolated from opium in 1832.^7,8^ Eventually, 20 pharmacologically active alkaloids in opium were reported.^3^ Codeine remains the most commonly used opioid derivative today, either alone or combined with dextromethorphan as an antitussive.^9^

Facilitating the administration of opioids, Irish physician Francis Rynd introduced the hollow needle in 1844^10^ to treat a patient's acute facial pain. In an account of the successful operation, Rynd wrote that the patient's pain ceased within a minute of the treatment.^11^ French physician Charles-Gabriel Pravaz introduced the piston syringe in 1853.^12^ Pravaz's syringe was designed for injecting coagulant into aneurysms, but it was modified and used for the administration of drugs. Scottish physician Alexander Wood used the hypodermic syringe to inject morphine hydrochloride into patients, and he noted a remarkably rapid effect of the treatments.^12^ These devices made controlled dosing of drugs, including opioids, possible.^1^

The inhalational agents of that era—ether and chloroform—were irritating and unpleasant. The stormy induction and agitation were mitigated by intramuscular morphine, and morphine was widely used as a premedicate, smoothing induction and enhancing the patient experience with anesthesia. Between 1895 and 1905, clinicians pushed the dose of morphine higher and higher (exceeding a dose of 2 mg/kg) and then combined it with scopolamine in an effort to provide total anesthesia. Not surprisingly, in an era with no mechanism to provide assisted ventilation for respiratory depression, the technique was abandoned.^13^ Ether was not an ideal inhalation agent because of its slow onset and the side effects of nausea, vomiting, and respiratory depression. Chemists were searching for a better drug.^14^

Charles Romley Alder Wright, an English chemist and physicist, synthesized diacetylmorphine in 1894 and named it heroin. Twenty-three years later, a European company (the forerunner of Bayer) brought heroin into clinical medicine. Heroin was marketed as a nonaddictive congener of morphine and was recommended to treat opioid addiction.^15^ In 1962, a comparative study of the analgesic potency on patients recovering from major thoracic, abdominal, or orthopedic surgery found that for acute pain, heroin was 2 to 4 times as potent as morphine when measuring the relief of “moderate, severe, or very severe postoperative pain” but also found it to be more addictive.^15,16^

## THE 20TH CENTURY AND BALANCED ANESTHESIA

The introduction of what came to be referred to as *balanced anesthesia* renewed the interest in opioids as an anesthetic base. Balanced anesthesia combines intravenous and volatile anesthetics to achieve amnesia, analgesia, unconsciousness, and muscle relaxation. John Lundy at the Mayo Clinic suggested intravenous morphine sulfate to smooth the induction of anesthesia with a volatile gas and had an influence on the preoperative, intraoperative, and postoperative anesthetic experience. Lundy coined the term balanced anesthesia and recommended the technique. He first published in the *Minnesota Medical Journal* in 1926, and then in the *Journal of the American Medical Association* in 1931.^17,18^ Prior to Lundy's publications, intravenous agents had been avoided, and the anesthetic depended wholly on anesthetic gas.

In 1910, before Lundy published his work, George Crile had published the theory of *anoci-association*: employing a light depth of anesthesia combined with intravenous local anesthesia injected into the surgical site.^19^ Later, with the resurgence of regional anesthesia and neuraxial blocks, these two anesthetic techniques were introduced into balanced anesthesia.

Other opioids became available—hydromorphone in 1924^20^ and hydrocodone in 1943^21,22^—and were used preoperatively and for postoperative analgesia but were not administered during anesthesia for a procedure.

## WORLD WAR II AND OPIOID ANESTHESIA

World War II provided an impetus to expand opioid therapy to include synthetic opioids. As early as 1932, Germany was preparing for war and stockpiling drugs. The country's morphine supplies were imported from other countries, so the supply pipeline would be at risk. Germany would need another mechanism of supply to treat the wounded and turned to synthetic opioids as a solution. German chemists synthesized meperidine (called pethidine in Europe) and found it to be an effective analgesic with or without anesthesia.^23^ Meperidine was later combined with nitrous oxide and curare, the first neuromuscular blocker.^24,25^

**Figure 1.**
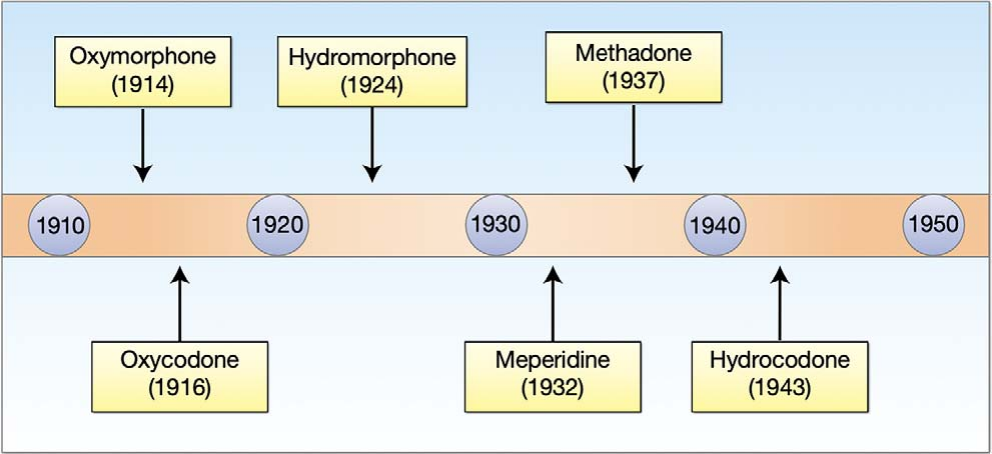
Historic development of synthetic opioids by German scientists.

The Germans were not alone in the opioid synthesis initiative, but they did take a different approach from other investigators. Most chemists concentrated on the modification of morphine, codeine, or thebaine, all of which can be extracted from opium. The Germans judged the morphine chemical structure to be too difficult to deal with and concentrated on the piperidine ring from which they developed meperidine. Between 1914 and 1943, focused and intensive research resulted in the development of an impressive array of new opioids (Figure 1).^1,2^

At the end of World War II, the Allies confiscated the German patents, including the patents for synthetic opioids. By that time, European physicians were used to intravenous anesthetic agents, while physicians in the United Kingdom and the United States depended on inhalational anesthetic agents. A factor contributing to this dichotomy was the anesthesia machine, an item too expensive to purchase in postwar Europe but common in the United States and the United Kingdom. Beginning with meperidine and moving in later years to other synthetic opioids such as fentanyl, using opioids to achieve anesthesia with lower alveolar gas concentrations of inhalational anesthetics became the norm.^26,27^

The European focus on intravenous anesthetic agents continued through the decade of the 1940s and the early 1950s. European investigators were in search of a better anesthetized state with smooth induction and emergence. For example, in 1954, Laborit and Huguenard introduced the concept of *artificial hibernation*, which involves applying hypothermia and adrenolytic agents until a specific therapy is administered.^28^ These investigators and others combined meperidine with other agents such as tranquilizers, benzodiazepines, and haloperidol. Haloperidol was a new tranquilizer, a butyrophenone, synthesized by Belgian physician Paul Janssen.^29^ When combined with phenoperidine, a new narcotic also developed by Janssen, the result was a pain-free, detached state with hemodynamic stability. Using the same molecule as a base, Janssen synthesized droperidol and then combined it with an even more potent opioid of his design—fentanyl. The combination of droperidol and fentanyl with nitrous oxide inhalation was termed *neuroleptanalgesia*.^30^

During the same era, opioid agonists, opioid antagonists, and opioid agonist-antagonists were being developed. German pharmacologist Julius Pohl manufactured the first antagonist in 1914 while trying to improve the analgesic effect of codeine.^15,31,32^ Pohl's compound, N-allylnorcodeine, was found to block the respiratory depression caused by morphine, and this effect was discovered 26 years later when McCawley and co-workers isolated nalorphine (N-allylnormorphine). In 1942, Weijlard and Erickson introduced nalorphine as a drug that would block the unwanted side effects of morphine and discovered that the drug hindered all the actions of morphine. Nalorphine had analgesic properties but only at high doses with unwanted psychotomimetic side effects.^15,31,32^ Next came naloxone, synthesized in 1960, a more potent opioid antagonist with minimal or no side effects.^33^ In response to the current opioid epidemic in the United States, naloxone is carried by first responders and stocked in treatment centers and college dormitories to reverse potent opioids causing respiratory depression and death.

## THE STORY OF FENTANYL AND ITS CONGENERS

After developing the butyrophenones (haloperidol and droperidol), Paul Janssen turned his attention to the synthetic opioids. He took a page from the earlier German investigators who focused on meperidine and the piperidine ring, which he surmised was important in inducing analgesia. Understanding the chemical approaches to building on the piperidine ring is important to understand the current opioid issue.

Whereas the morphine molecular structure is based on a phenanthrene ring and is found in plants, meperidine is based on a much simpler piperidine ring. With the goal of discovering a more soluble compound than morphine, the Janssen researchers replaced the methyl group attached to the N on the meperidine molecule with a benzene ring.^34^ They then added a C=O to the newly placed benzene ring and found that it increased analgesic potency. Changing the C=O to a C-OH further increased the potency of the analgesia. This compound was named phenoperidine and was the precursor of fentanyl. Further modification of the molecule led to the synthesis of sufentanil, alfentanil, and carfentanil.^34^ Each derivation altered potency, onset, duration, and offset. The pharmaceutical company Glaxo Wellcome Inc. developed remifentanil, but the drug was deemed a financial failure because of two characteristics: it was an analgesic on/off switch, resulting in immediate pain when the infusion was interrupted, and it had the propensity for hyperalgesia postprocedure.^34-36^ Glaxo Wellcome sold the patent for remifentanil to Abbott Laboratories.^35,36^

Within 3 years of synthesis, fentanyl was released in Europe but not in the United States. Robert Dripps, Chair of Anesthesiology at the University of Pennsylvania, vehemently objected to US Food and Drug Administration (FDA) approval on the grounds that the drug was too potent, caused chest wall rigidity requiring tracheal intubation and mechanical ventilation, and had increased potential for abuse compared to morphine.^15,37^ In terms of abuse potential, Dripps's concerns have been validated.

Fentanyl became available in the United States in 1968 but only in combination with droperidol in a 50:1 ratio.^15^ This combination was suggested by the work of George de Castro in Belgium; it was marketed as Innovar.^15^ The drug failed in the United States, principally because of the dose of droperidol that was at least 4 times the recommended dose. When the FDA finally approved fentanyl, it was released only as a single vial of 50 μg in 1 cc, and its usage was kept under these strict constraints for another 6 years.^15^ However, the illicit production and abuse of synthetic opioids increased and led to a serious epidemic, with the mortality from synthetic opioids, primarily fentanyl, rising to more than 31,000 deaths between 2013 and 2018.^38^ According to the US Drug Enforcement Administration, 67% of all drug overdoses in 2021 were attributable to synthetic drugs such as fentanyl, and the agency urged the federal government to increase fentanyl visibility by dedicating May 10, 2022 as National Fentanyl Awareness Day.^39^

The synthesis of fentanyl demonstrates an important reality. The chemical formulation, although tedious to develop and test, is relatively simple, easy to make, and inexpensive. The illegal entry of fentanyl into North America from China corroborates the early warnings of high potency, respiratory depression, and potential for abuse.

## HIGH-DOSE OPIOID ANESTHESIA

Opioids (morphine) were not used as primary agents in anesthesia by 1915, with broader acceptance of inhalational agents, because of the lack of adequate monitoring devices for vital signs and the necessity of patient admission to the hospital.^1^ Despite this deviation, twilight sleep, which had previously been accomplished using low doses of opioids, reemerged after World War II as sedation-analgesia or monitored anesthetic care in which the goal is hypnosis, amnesia, and analgesia with the patient still able to respond and follow commands.^1^ In the 1960s, a conflation of events in pharmacology, technology, and clinical medicine resulted in the reentry of opioids in high doses for anesthesia in cardiac patients.^40^ Curare was isolated and studied, resulting in a new class of drugs (the neuromuscular blockers) that became available for neuromuscular paralysis, as did controlled oxygen therapy.^40^ The most important change in anesthetic practice was the cuffed endotracheal tube combined with mechanical ventilators. The ability to manage respiratory depression, the most serious side effect of high-dose opioids, led clinicians around the world to reevaluate their use of high-dose opioid anesthesia.

In the United States, the cardiac anesthesia group at Massachusetts General Hospital was dealing with high mortality among patients with rheumatic aortic and mitral valve disease. Patients came to surgery with low cardiac output and cardiac index, pulmonary hypertension, and increased extravascular lung water.^41^ The standard anesthetic induction techniques used intravenous thiopental and succinylcholine, with maintenance of anesthesia via halothane, nitrous oxide, curare, and oxygen. The result in these compromised patients was hemodynamic instability, arrhythmia, and, in some cases, death.^41^ The patients who survived the operation went to the intensive care unit (ICU) intubated, ventilated, and sedated with morphine. The anesthesia team noted that while patients were on the ventilator, very high doses could be achieved with incremental dosing, and the patients remained hemodynamically stable. They reasoned that the standard induction of anesthesia could be replaced with an oxygen-morphine technique in which patients breathed 100% oxygen while a slow infusion of morphine at 3 mg/kg was initiated. When the patient lost consciousness, a neuromuscular blocker was administered, and the trachea was intubated. This approach resulted in no change in hemodynamics. The group reported outcomes for 15 patients, 7 with aortic valve disease and 8 normal controls.^41^ Surprisingly, the patients with aortic valve disease showed improved cardiac function, with improvement in cardiac output and lower systemic vascular resistance. Up to 3 mg of morphine was given intravenously during the procedure.^41^

In Brussels, de Castro and Viars reported using high-dose fentanyl for routine procedures such as colorectal surgery and cholecystectomy.^42^ Relatively healthy patients undergoing routine surgical procedures received fentanyl, a relatively new drug at the time, instead of morphine. Several benefits came with using fentanyl vs morphine. Fentanyl is more potent than morphine; has a faster onset and shorter duration of action; does not release histamine, a consistent side effect of morphine; and does not cause vasodilation. Additionally, with fentanyl, patients spent less time on a ventilator postoperatively and were able to be extubated and return to spontaneous ventilation with a native airway.^43^ For these reasons, the use of high-dose fentanyl would eventually replace morphine in the United States by the 1970s.

## THE FUTURE OF OPIOIDS IN ANESTHETIC MANAGEMENT

Since the late 1980s, high-dose fentanyl anesthesia (50-100 μg/kg)^44^ has not been necessary with the advancements in anesthesiology. Advances in induction agents, such as propofol; neuromuscular blockers, such as succinylcholine and rocuronium; and the insoluble inhalational agents, such as nitrous oxide, replaced the use of high-dose fentanyl.^45,46^ Lower narcotic dosing has resulted in faster and smoother emergence from anesthesia. Intravenous agents that became available have greatly improved perioperative pain management: acetaminophen, ibuprofen, ketorolac, subanesthetic doses of ketamine, and continuous infusions of low-dose lidocaine and dexmedetomidine. The introduction of these opioid substitutes has allowed for lower dosages of opioids, both during and after surgery, and has relieved many opioid-related postoperative complications.^47^ Altogether, the use of these agents has made opioid-free anesthesia achievable and affordable.

In 2018, we published an evidence-based set of anesthetic guidelines for opioid-free anesthesia for procedures lasting more than 2 hours, using low-dose continuous infusions of lidocaine and dexmedetomidine, intermittent intravenous dosing of acetaminophen, and ½ minimum alveolar concentration of isoflurane.^48^ Our cost analysis showed a reasonable total cost for administered drugs, less than the cost of one intravenous dose of acetaminophen and less than the cost of one dose of liposomal bupivacaine.^49^ A cost is associated with drugs being continuously infused because of the requirement for an infusion device that delivers reliable and precise dosing.

Envision an operating room table or ICU bed with the patient centered in the room. To the right of the patient is an anesthesia machine with a ventilator, delivering the selected dose of an inhalational agent. To the left of the patient is a second anesthesia machine delivering intravenous anesthetic agents, each with its own controller. The summation of this multimodal approach can be seen by examining the patient, the monitor positioned over the patient on the wall or ceiling and the data displayed on the anesthesia machine monitor.

An explosion of information has resulted from the availability of these continuous infusion devices rather than intermittent bolus dosing.^50^ Studies have addressed the pharmacokinetics and pharmacodynamics of opioids,^51^ opioid selection,^52^ and recovery from intravenous narcotics.^53^ We are now aware of context-sensitive half-times of drugs that are continuously infused, leading to improved dosing.^54^ In terms of monitoring during anesthesia, we now have the technology to measure depth of anesthesia.^55,56^ Technology and information flow will continue to improve to the betterment of patient safety and the patient experience.^55,56^

## THE NEUROBIOLOGY OF ADDICTION

**Figure 2.**
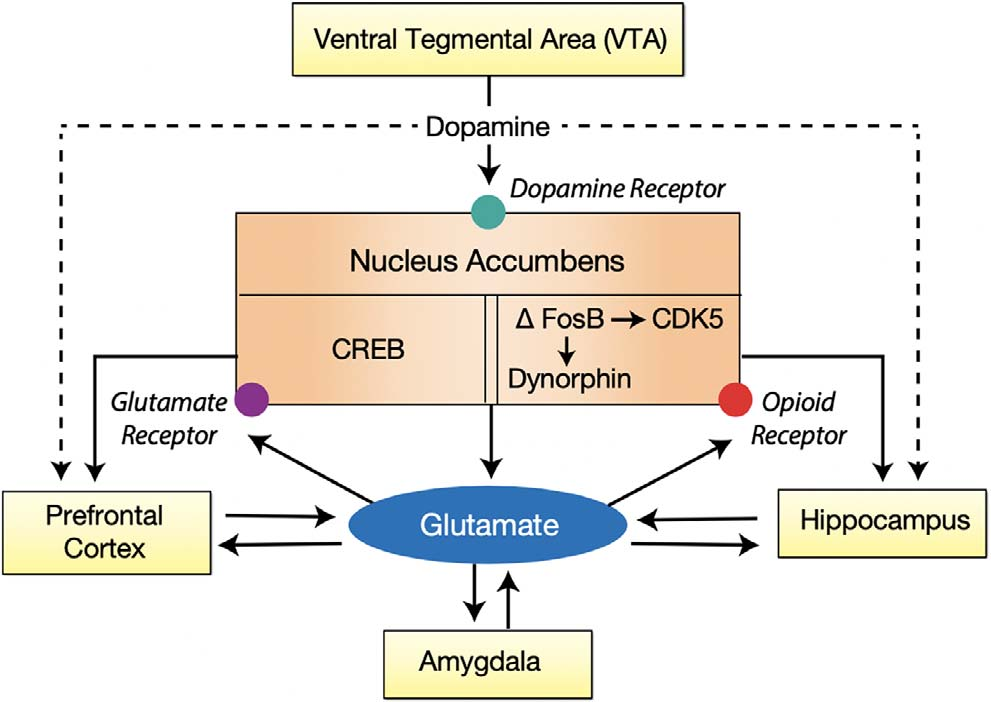
**The molecular biology of addiction.** CDK5, cyclin-dependent kinase 5; CREB, cAMP response element-binding protein; ΔFosB, a transcription factor.

**Figure 3.**
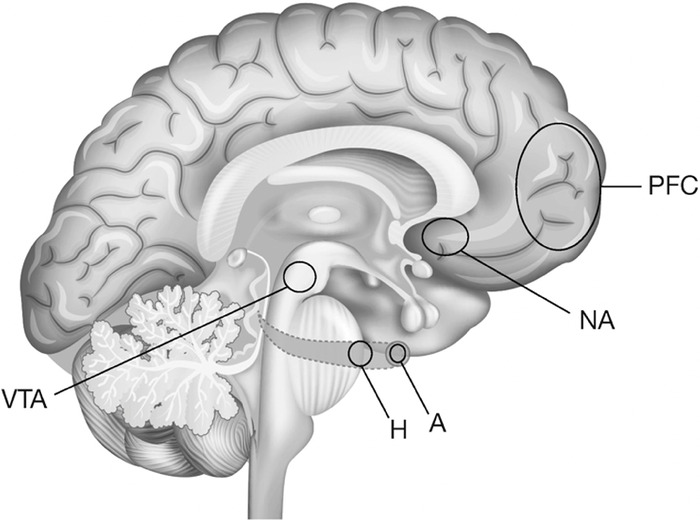
**The anatomic final common pathway to addiction.** A, amygdala; H, hippocampus; NA, nucleus accumbens; PFC, prefrontal cortex; VTA, ventral tegmental area.

The neurobiology of addiction is an intense area of research. A brain reward circuit was demonstrated in rodents. Rats taught to push a lever to self-administer addictive drugs (heroin, cocaine, amphetamines) would begin to show addictive behavior, much like addicted humans and would not eat or sleep, exhibited excessive lever activation, and manifested other behavioral traits of addiction.^57^ When the drug was withdrawn from the environment, the pleasure associated with the drug administration was not forgotten but rather remained intact for months, resulting in reactivation and craving in response to cues of addiction or stress. Subsequently, newer research techniques employing functional imaging and gene sequencing confirm a final common pathway for all types of addiction.^57,58^ The anatomic sites of the mesolimbic dopamine system in the brain that make up this reward/addiction circuit—the ventral tegmental area (VTA), nucleus accumbens (NA), prefrontal cortex, amygdala, and hippocampus—are depicted in Figures 2 and 3.^57-60^

The communication between these sites has a molecular basis. When stimulated, the neurons of the VTA release dopamine to the synaptic clefts and receptors of the NA. In turn, the NA communicates with the prefrontal cortex, amygdala, and hippocampus.^61^ When dopamine is released by the VTA to the NA, the result is the release of two transcription proteins. The cAMP response element binding protein (CREB) rises immediately. Exposure to an addictive drug regulates gene expression to produce dynorphin. Dynorphin is a natural molecule with opioid-like effects that in turn inhibit neurons in the VTA.^58^ This inhibitory effect on the reward circuit results in tolerance to the stimulating drug.^62^ However, the production of CREB and the biologic effects last only a matter of days. Another transcription factor, DeltaFosB, is slowly elaborated and rises to a peak level that will last for months.^58^ This transcription factor induces anatomic changes, mainly a dendritic change in the NA, resulting in a bushy appearance in photomicroscopy that parallels the intransigence of addictive behavior and explains the tendency to relapse following often extended periods of abstinence. Activation of the reward circuit begins when the VTA is stimulated to release dopamine to the NA, although the NA also has opioid receptors that can be directly stimulated.^63^ The neurotransmitters activate the amygdala to assess whether the experience is pleasurable or not and what cues are associated with that experience.^64^ The hippocampus records the memory of that experience,^65^ and the prefrontal cortex processes the information to determine ultimate behavior.^66^ Glutamate is the transcription factor to complete communication.^67^

Certain individuals have a genetic predisposition to addiction, nearly 50% as proposed by the National Institute on Drug Abuse, making genetic screening a possible predictor of future opioid addiction.^68^ Epigenetics, a relatively new field of study involving environmental effects on DNA modifications, is an avenue that can improve the prediction of drug addiction.^69^ DNA screening for possible addiction markers has the potential to prevent individuals at risk for addiction being overdosed or overprescribed. Screening mechanisms, like the Opioid Risk Tool that assesses social determinants of health to predict the risk of opioid abuse and misuse, could be enhanced by the study of epigenetics.^70^

In addition, anesthetic and analgesic drugs are now available to manage pain in perioperative and periprocedural patients with an opioid-sparing or ultimately an opioid-free regimen. We now have at our disposal the nonsalicylate, nonopioid analgesics and nonsteroidal anti-inflammatory drugs (eg, acetaminophen and ibuprofen) and the ability to design a multimodal approach to anesthesia that lessens the need for opioid analgesia. The basic science of neurobiology will provide new methods of therapy and intervention and will enhance our understanding of addiction and help prevent new persistent opioid abuse.

## CONCLUSION

With advances in medical research, common opioids have been synthetically engineered to manipulate their pharmacokinetic properties to enhance their curative/healing potential. The use of opioids as anesthetic agents in the United States for the management of pain has led to a growing dependence on opioids among patients undergoing surgical care. Deaths from drug overdose have skyrocketed since the 1990s, and drug overdose is now one of the leading causes of death in the United States. Deaths from opioid overdose (especially fentanyl) has become such a major health crisis in the United States that May 10 has been recognized as National Fentanyl Awareness Day by the DEA to spread awareness about the dangers of fentanyl. Analgesic drugs that have a high risk of addiction should only be prescribed for a limited period and if proven ineffective in that time frame, an alternative drug should be tried. Additional pharmacogenetic research in the field of addiction and neurobiology of opioid interactions will provide a better approach to treating patients at risk for opioid abuse and dependence. Going forward, opioid-sparing improvements in analgesia and anesthesia with better technology for monitoring, new pharmaceutical compounds, and using genetic markers for addiction will enhance our understanding of addiction and help prevent new persistent opioid abuse.

## Notes

*This article meets the Accreditation Council for Graduate Medical Education and the American Board of Medical Specialties Maintenance of Certification competencies for Patient Care and Medical Knowledge.*
